# Diagnosis of Mondor’s Disease in the Setting of Right-Sided Anterior Chest Wall Pain

**DOI:** 10.7759/cureus.31894

**Published:** 2022-11-25

**Authors:** Matthew Warner, Muhammad Durrani, Varsha Yerram, Andrew Coppa, Andrew Barra

**Affiliations:** 1 Emergency Medicine, Inspira Medical Center, Vineland, USA

**Keywords:** point-of-care ultrasound, clinical examination, clinical case report, superficial thrombophlebitis, mondor’s disease

## Abstract

A 60-year-old male patient presented to the emergency department of our hospital with right-sided chest wall pain and a palpable subcutaneous cord-like structure along the right anterior chest wall. Examination revealed tenderness over the cord-like structure, and the skin overlying the structure was freely mobile and did not have any sign of infection or inflammation. Bedside ultrasonography revealed an uncompressible tubular structure with the absence of a color Doppler flow signal. The patient’s presentation was suggestive of Mondor’s disease. The patient was discharged with instructions to utilize anti-inflammatory drugs, perform warm compresses, and seek primary care follow-up to ensure resolution. Mondor’s disease is a rare disorder characterized by a superficial thrombophlebitis of the subcutaneous veins of the chest wall. For its diagnosis, a thorough examination of the patient’s medical history and physical condition is suggested; further, the performance of point-of-care ultrasonography has also been suggested. Once recognized, further emergency department workup is typically unnecessary in cases of primary Mondor’s disease. Despite being a mostly self-limited condition, greater awareness of this rare disease entity is required to ensure and coordinate close outpatient follow-up as well as monitor resolution due to its association with secondary causes such as vascular and breast carcinoma, vasculitis, and hypercoagulable disorders.

## Introduction

Mondor’s disease is a form of superficial thrombophlebitis of the subcutaneous veins of the thoracoabdominal and genitourinary regions. It is a rare clinical disorder that most commonly affects the anterolateral thoraco-abdominal wall but may also be found in the penile and axillary regions [1]. The true incidence of Mondor’s disease is unclear; however, case series have estimated an incidence rate of 0.07-0.96% on the chest wall, and less than 400 cases have been reported in the medical literature [2,3]. There is a female-to-male predominance, and the average age of diagnosis is between 30 and 60 years [4]. Mondor's disease is idiopathic in 50-60% of the reported cases, but multiple case series have suggested an association between local trauma and Mondor's disease, as well as multiple secondary causes [4]. Typically, examination reveals a cord-like palpable structure in a location that is dependent on which subcutaneous vein is affected. The thoracoepigastric vein is most commonly affected [4]. The palpable cord-like structure often appears to be longitudinal along the anterolateral thoraco-abdominal wall due to the vertical orientation of the venous network [1]. The skin overlying the cord-like structure is usually mobile and elastic without infectious or inflammatory changes. Mondor's disease is primarily a clinical diagnosis, and point-of-care ultrasonography is emerging as an effective and rapid method for confirming its diagnosis.

## Case presentation

**Figure 1:**
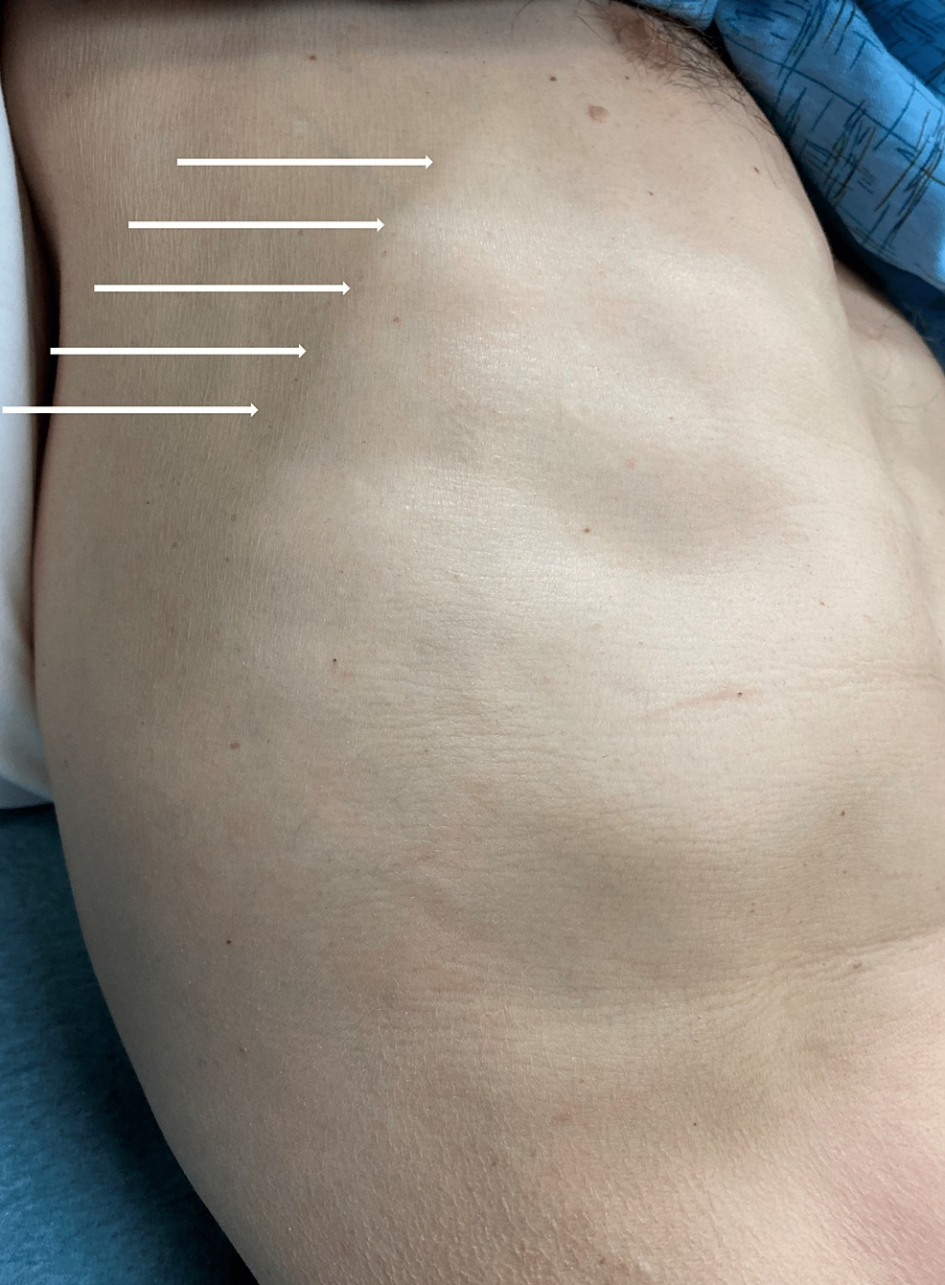
A palpable, longitudinal cord-like structure (white arrows) on the right anterolateral chest wall consistent with Mondor's disease.

A 60-year-old male patient without a significant past medical history presented to the emergency department of our hospital with right-sided anterior chest wall pain. The patient noted a tender longitudinal band over his right anterior chest wall shortly after working underneath his car. He denied any experience of trauma, recent surgical interventions, or recreational drug abuse. Additionally, he denied any familial history of malignancies, autoimmune diseases, or coagulopathic disorders. His symptoms were exacerbated with palpation of the cord-like structure. Vital signs were within normal range, and examination revealed a tender, palpable subcutaneous cord on the chest wall (Figure 1). The skin overlying the structure was freely mobile and did not possess any sign of infection or inflammation. Point-of-care bedside ultrasonography confirmed an uncompressible hypoechoic venous tubular structure with the absence of a color Doppler flow signal. The patient’s presentation of superficial thrombophlebitis of the subcutaneous veins on his chest wall was consistent with Mondor’s disease. He was discharged with instructions to use non-steroidal anti-inflammatory medications, apply warm compresses, and seek close follow-up with his primary care physician. Over the next four to six weeks, he noted improvement in his pain and the resolution of the cord-like structure.

## Discussion

Mondor's disease is a rare clinical condition and suffers from a lack of clinical awareness as a result. It is important to distinguish primary (idiopathic) and secondary causes after a presumptive diagnosis. Underlying conditions that have been associated with secondary Mondor's disease include vascular and breast malignancies, vasculitis, hypercoagulable disorders, and post-surgical states [4]. The diagnosis is clinical, and definitive testing and work-up for underlying causes in the emergency department are not necessary as long as they can be safely performed on an outpatient basis using a multi-modal approach. The use of point-of-care bedside ultrasonography presents a unique opportunity for the clinician to rapidly augment their physical examination and confirm the diagnosis. Ultrasonographic features consistent with Mondor’s disease include noncompressible veins with a lack of venous color Doppler flow. Early recognition may allow for the reduction of unnecessary diagnostic testing, imaging, and costs to the patient in the emergency department. Additionally, it will allow the emergency physician to better inform their patients of the secondary causes of Mondor's disease and emphasize the importance of outpatient follow-up. Once diagnosed, treatment comprises using non-steroidal anti-inflammatory medications and warm compresses. Mondor's disease is a self-limited illness that often spontaneously resolves within four to eight weeks [1]. There are some existing recommendations regarding the use of anticoagulation in Mondor's disease, but they remain controversial at this time [1]. Although a rare condition, clinical awareness of this disease entity is crucial for the emergency physician. We hope this case report adds to the body of literature on Mondor’s disease and reinforces the importance of point-of-care ultrasonography in confirming its diagnosis.

## Conclusions

Our patient presented with clinical features indicative of Mondor’s disease. Although the diagnosis was suspected based on physical examination, the utilization of point-of-care ultrasonography allowed for rapid confirmation of the diagnosis and augmented the physical examination. Awareness of this disease entity and the use of point-of-care ultrasonography helped the patient avoid a potentially costly and lengthy hospital stay. The patient was discharged with instructions to use anti-inflammatory drugs and warm compresses and obtain primary care follow-up to ensure resolution. Over the subsequent four-to-six-week follow-up period, the patient noted improvement in his symptoms and the resolution of the cord-like structure.
